# Are Stakeholder Prioritized Post-Acute Care Practices Documented and Do They Improve Outcomes?

**DOI:** 10.1093/geroni/igab046.2280

**Published:** 2021-12-17

**Authors:** Natalie Leland, Stephanie Rouch, Elizabeth Skidmore

**Affiliations:** University of Pittsburgh, Pittsburgh, Pennsylvania, United States

## Abstract

The receipt and intensity of rehabilitation services, such as occupational and physical therapy, have been associated with lower risk of readmissions. Yet, little is known about the care. This study quantified the frequency of documented post-acute care (PAC) stakeholder-prioritized practices and their associations with hospital readmissions. A PAC stakeholder advisory board (e.g., physicians, rehabilitation providers across settings) prioritized key practices to evaluate. Medicare claims and electronic medical records were used to construct an episode of care for patients age 65 or older. Eligible patients were discharged from one of nine acute hospitals to a PAC setting (i.e., inpatient rehabilitation, skilled nursing, home health) within one large health system between August 2016 and August 2018. Descriptive statistics characterized the cohort and frequency of documented practices. Logistic regression examined associations among the practices and readmissions, by setting. Stakeholders prioritized (a) education, (b) cognition assessment and treatment, and (c) medication management. Among these PAC patients (n=3,227) there was variation in documentation for each practice by setting. Documentation of medication management at any point during the stay ranged from less than 1% to 54% of patient stays among settings. There was a significant relationship between the practices and readmissions. Within inpatient rehabilitation, every additional day patient and caregiver education was documented by occupational therapy was associated with 21% lower odds of readmission (p<0.05). This study highlights the variability in documentation of stakeholder-prioritized practices across PAC and their associations with readmissions. Future work is needed to enhance the systematic delivery and documentation of these practices.

